# Site‐Specific Mutations on *KRAS* , *NRAS* , and *BRAF* Corelate With the Frequency of ctDNA in Colorectal Cancer

**DOI:** 10.1002/cnr2.70292

**Published:** 2025-07-23

**Authors:** Fumihiro Yoshimura, Yoichiro Yoshida, Teppei Yamada, Keita Tanaka, Takaomi Hayashi, Hideki Shimaoka, Ryohei Sakamoto, Naoya Aisu, Gumpei Yoshimatsu, Suguru Hasegawa

**Affiliations:** ^1^ Department of Gastroenterological Surgery Fukuoka University Faculty of Medicine Fukuoka Japan; ^2^ Department of Medical Informatics and Digital Medicine Fukuoka University Hospital Fukuoka Japan

**Keywords:** *BRAF*, circulating tumor DNA, colorectal cancer, *KRAS*, liquid biopsy, *NRAS*

## Abstract

**Background:**

Early prediction of metastatic risk after tumor resection for colorectal cancer (CRC) is critical to improve treatment outcomes. Although circulating tumor DNA (ctDNA) is an important biomarker in CRC patients, positivity is variable because cutoff values for each gene have not been clearly established. When examining the mutant allele frequency (MAF) of a gene, the cutoff value is the same for the same gene, even if the mutation sites are different. In this study, we examined the relationship between MAF and the genetic mutation site and factors that influence the prediction of recurrence by ctDNA.

**Methods:**

This study included 422 CRC patients who underwent surgery. ctDNA was sampled from blood samples of 102 CRC patients with *KRAS, NRAS*, and *BRAF* mutations and analyzed using the digital polymerase chain reaction system. Preoperative, postoperative day 1, postoperative day 7, and postoperative day 30 MAF were examined for each gene mutation site.

**Results:**

Kruskal–Wallis test revealed significant differences in MAF between mutated codon sites at all MAF assessment times (*p* < 0.001). The MAF values of *KRAS* codon 146 at all time points were significantly higher than for the other mutation sites. Steel‐Dwass tests revealed *KRAS* codon 146 had significantly higher MAF values than *KRAS* codons 12 and 13 on all blood collection dates. Similarly, *BRAF* codon 600 had significantly higher MAF values than *KRAS* codon 12 on all blood collection dates.

**Conclusions:**

This study revealed that MAF values differed significantly depending on the site of mutation, even for the same gene. These results suggest that MAF cutoff values may need to be established for each gene mutation site.

## 1 Introduction

Colorectal cancer (CRC) is one of the most common cancers, affecting more than 1.8 million patients worldwide annually [1]. Treatment for non‐metastatic CRC consists of curative resection and adjuvant chemotherapy, depending on the stage of the cancer. Despite improvements in surgery and chemotherapy, the 5‐year mortality rate of CRC remains high at approximately 40% [2]. Early prediction of the risk of metastasis after tumor resection is essential to improve clinical outcomes.

Kirsten rat sarcoma viral oncogene homolog (*KRAS*) mutations are detected in 40%–50% of CRC patients, and neuroblastoma RAS viral oncogene homolog (*NRAS*) and v‐raf murine sarcoma viral oncogene homolog B (*BRAF*) mutations are detected in 1.2%–8.5% [3, 4, 5]. Mutations in *KRAS* and *NRAS* lead to continued activation of the epidermal growth factor receptor (EGFR) pathway and promote tumor growth and survival, even with pharmacological blockade of EGFR [6]. *BRAF*, a serine/threonine protein kinase, plays an important role in the EGFR‐mediated mitogen‐activated protein kinase pathway, which is activated by the *RAS* small GTPase [7]. Many genetic mutations are known in CRC, but *KRAS*, *NRAS*, and *BRAF* are very important genes that are directly related to treatment.

Tissue biopsy is currently the standard method for diagnosis and biomarker detection for all cancers. However, tissue‐based genetic testing cannot precisely and accurately reflect tumor status because of clonal evolution over time and heterogeneity within tumors [8]. In recent years, circulating tumor DNA (ctDNA) has gained recognition as a highly sensitive biomarker for various types of cancer [9, 10, 11, 12]. Analysis of ctDNA from liquid biopsies such as blood and urine has made it possible to comprehensively characterize cancer genomes [13, 14, 15, 16]. Furthermore, liquid biopsy can be easily repeated and is less invasive compared with tumor biopsy [17]. However, while liquid biopsy has shown great promise for a variety of purposes in some tumor types, it has only been validated for limited applications in clinical practice [18]. We previously reported on liquid biopsy targeting *KRAS* or *BRAF* mutations in patients with CRC [19, 20]. It was difficult to predict recurrence by analyzing *KRAS* and *BRAF* mutations alone. However, our analyses excluded patients with two mutations and did not analyze *NRAS* mutations. A total of 57 phase II/III trials focused on ctDNA in the detection of minimal residual disease have been identified, with a marked increase in their initiation in recent years [21]. Trial methods vary, and currently no clear method has been established. Furthermore, ctDNA as a longitudinal marker has not yet been validated.

Digital polymerase chain reaction (dPCR) is effective in analyzing genetic mutations because of its ability to detect and quantify mutations in small amounts of DNA with high sensitivity [22]. Nakamura et al. reported that preoperative detection of the *KRAS* mutation in ctDNA is an independent risk factor for recurrence of CRC [23]. A systematic review concluded that in patients with mutant *KRAS* colorectal cancer, plasma *KRAS*‐positive status may be a poor prognostic factor for overall survival, progression‐free survival, and disease‐free survival [24]. However, few patients with stage III or lower disease were included. Notably, the cutoff values of genes and mutation sites vary; in these studies, the cutoff values for ctDNA positivity differed, and the methods were not uniform. Compared to *KRAS*, there are currently few reports on the prediction of CRC recurrence after radical resection by perioperative ctDNA for *NRAS* and *BRAF* mutated genes. Additionally, no studies have examined whether there is a difference in the detection rate of ctDNA focusing on the genetic mutation site. Although ctDNA is an important biomarker in CRC patients, methods and cutoff values have not been clearly established.

The purpose of this study was to examine the relationship between ctDNA detection rates and *KRAS*, *NRAS*, and *BRAF* genetic mutation sites and the factors that influence the prediction of recurrence by ctDNA.

## 2 Methods

### 2.1 Study Design and Patients

This was a single‐center prospective study of patients who underwent surgery for CRC without distant metastasis at Fukuoka University Hospital from April 2018 to June 2020. A total of 422 patients diagnosed with primary colorectal cancer who underwent surgery were enrolled. For tissue *KRAS*, *NRAS*, and *BRAF* tests, the MEBGEN RASKET‐B kit (MBL, Tokyo, Japan), which applies the polymerase chain reaction‐reverse sequence‐specific oligonucleotide method, was used in accordance with the manufacturer's protocol [19]. We excluded cases with *KRAS* wild‐type, *NRAS* wild‐type, *BRAF* wild‐type, and minor *KRAS* mutations such as *KRAS*‐exon 3 p.A59T, *KRAS*‐exon 2 p.G12R, and *KRAS*‐exon 2 p.G13R. Mutations for which cases existed and for which a dual probe TaqMan assay was commercially available were included in the analysis. The Institutional Review Board of Fukuoka University Faculty of Medicine approved this research (U‐1909001, 2017M.35). Written informed consent was obtained from all patients. All procedures were performed in accordance with the Declaration of Helsinki.

### 2.2 Blood Collection

Peripheral blood samples were collected preoperatively and on postoperative days 1, 7, and 30 using BD Vacutainer PPT plasma preparation tubes (Becton, Dickinson and Company, Franklin Lakes, NJ, USA) as described previously [19]. Blood samples were centrifuged at 1100 × g for 10 min at 4°C within 2 h after collection. Plasma was transferred to microtubes and stored at −80°C until use.

### 2.3 ctDNA Extraction From Plasma Samples

The plasma samples were recentrifuged at 16000 × g for 10 min at 4°C to remove debris. ctDNA was extracted from 1.0 mL plasma using the Maxwell RSC cfDNA plasma kit (Promega Corporation, Madison, WI, USA) and Maxwell RSC Instrument (Promega Corporation, Madison, USA) following the manufacturer's protocol, as described previously [19].

### 2.4 Mutation Detection by dPCR

The quantity of ctDNA was calculated using the QuantStudio 3D Digital PCR System (Applied Biosystems, South San Francisco, CA, USA) as previously reported [19]. DNA amplification using the ProFlex PCR System (Applied Biosystems, South San Francisco, CA) was performed as previously described [17]. For dPCR, a dual probe TaqMan assay for *KRAS*, *NRAS*, and *BRAF* (ThermoScientific) was used. For *KRAS*, nine mutations were covered: exon 4 p.A146T, exon 4 p.A146V, exon 2 p.G12A, exon 2 p.G12C, exon 2 p.G12D, exon 2 p.G12S, exon 2 p.G12V, exon 2 p.G13D, and exon 3 p.Q61H. In *NRAS*, four mutations were examined: exon 2 p.G12D, exon 3 p.Q61K, exon 3 p.Q61L, and exon 3 p.Q61R. In *BRAF*, exon 15 p.V600E was covered. Results were analyzed using QuantStudio 3D Analysis Suite Cloud software. Automatic call assignment for each data cluster was manually adjusted as needed. Two independent investigators blinded to clinical information performed the dPCR data analysis. Assay results were reported as mutant allele frequencies (MAF), defined as the ratio of mutant DNA molecules to the sum of wild‐type and mutant DNA molecules. Plasmid DNAs harboring *KRAS*, *NRAS*, and *BRAF* with mutations (GeneArt, Thermo Fisher Scientific) were used to confirm the sensitivity of dPCR for each mutation as previously reported [19, 20].

### 2.5 Statistical Analysis

Statistical analysis was performed using IBM SPSS Statistics (IBM Japan Inc., Tokyo, Japan). Mann–Whitney *U* test was used to compare quantitative variables. The Kruskal–Wallis test is used to compare independent samples with equal or different sample sizes. If the Kruskal‐Wallis test showed a significant difference, the Steel‐Dwass test was performed to test between the two groups. *p*‐values of < 0.05 were considered statistically significant. The correlation coefficients and heatmap were derived using Python.

## 3 Results

### 3.1 Patient Characteristics

**FIGURE 1:**
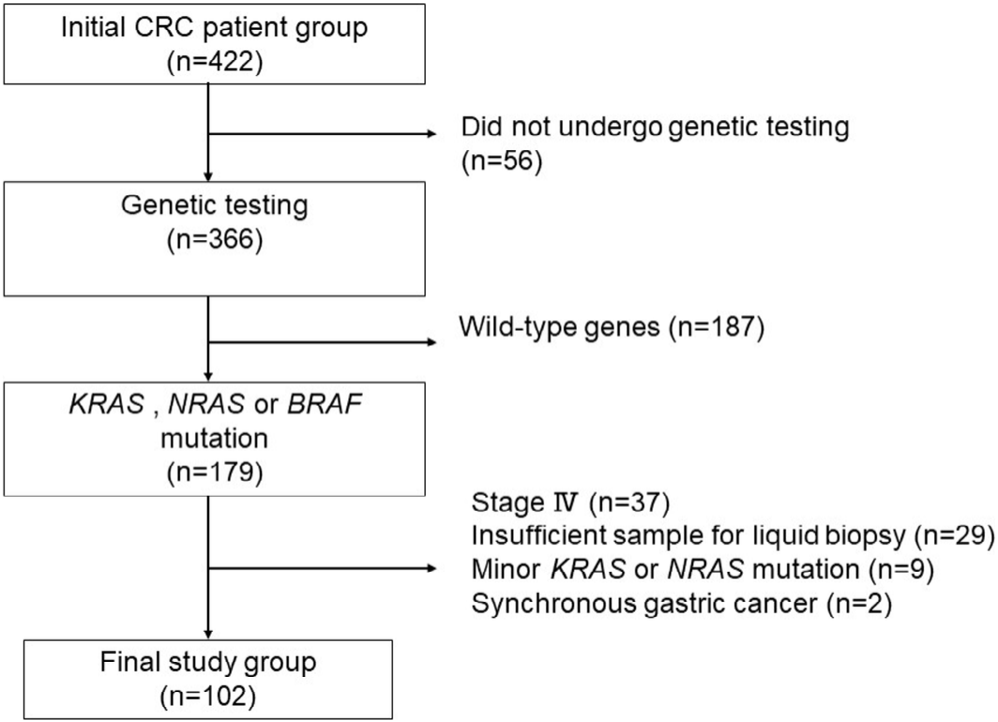
Flow chart for patient selection.

Of the 422 patients who received surgery for CRC at our institution from April 2018 to June 2020, 366 patients underwent genetic testing of resection specimens. Among the 366 patients, 179 patients had mutations in the *KRAS*, *NRAS*, and *BRAF* genes, while 187 patients did not harbor mutations in these genes. We further excluded 77 patients with distant metastases, minor gene mutations, synchronous gastric cancer, and insufficient liquid biopsy samples (Figure 1). Finally, 102 patients were included in the analysis.

The characteristics of the 102 CRC patients in the study group are listed in Table 1. The median age was 68 years (range, 28–90), and the median observation time was 36.5 months. The Eastern Cooperative Oncology Group performance score (ECOG PS) was 0 in 68 patients, 1 in 23 patients, and ≥ 2 in 11 patients. In this study, the codons covered by the MEBGEN RASKET‐B kit (MBL, Tokyo, Japan) were included in the analysis. The mean diameter of the primary tumor was 42.7 mm (range, 6–105 mm). Pathological tumor depth was less than T2 in 27 patients and T3 or greater in 57 patients. Regarding staging, 2 cases were stage 0, 24 were stage I, 38 were stage II, and 38 were stage III. Recurrence was observed in 25 cases, most frequently in the lungs, followed by the liver.

**TABLE 1:** Demographics and characteristics of patients with *KRAS, NRAS* or *BRAF* mutation (*n* = 102).

	*N* = 102
Sex	
Male/Female	56/46
Age (years)	68 (28–90)
BMI (kg/m^2^)	22.1 (14.2–41.0)
ECOG PS	
0/1/2 ≤	68/23/11
Tumor location	
Right‐sided/left‐sided	31/71
CEA (ng/mL)	8.2 (0.8–82.6)
CA19‐9 (U/mL)	14.6 (1.6–199)
*KRAS* mutation	
A146T/A146V/G12A/G12C/G12D/G12S/G12V/G13D/Q61H	5/3/6/2/34/7/16/13/3
*NRAS* mutation	
G12D/Q61K/Q61L/Q61R	2/1/1/1
*BRAF* mutation	
V600E	11
Tumor size (mm)	42.7 (6–105)
Histological grade	
Well/moderately/poorly/mucinous	76/16/2/8
p‐Tumor depth	
Tis/T1/T2/T3/T4 (T4a/T4b)	2/9/20/44/24 (22/2)
p‐Lymph node metastasis	
N0/N1 (N1a/N1b/N1c)/N2 (N2a/N2b)	64/29 (12/16/0)/9 (5/4)
p‐stage	
0/I/ll (lla/llb/llc)/lll (llla/lllb/lllc)	2/24/38 (27/10/1)/38 (6/25/7)
Recurrence	25
Lymph node/Liver/Lung/Peritoneum/Local	3/7/10/6/2

Abbreviations: BMI, body mass index; ECOG PS, Eastern Cooperative Oncology Group performance score.

### 3.2 Correlations of Clinicopathological Factors

**FIGURE 2:**
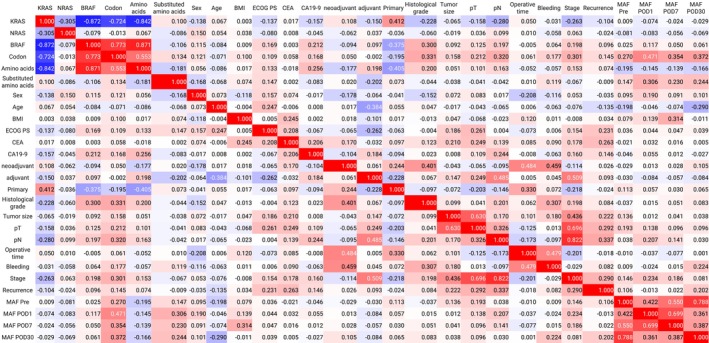
Heat map of correlation between MAF and clinicopathological factors. The heat map is created so that a correlation coefficient of 1 is red and −1 is blue. The numbers in the figure represent correlation coefficients. MAFs with different dates of blood sampling correlated with each other; otherwise, only mutated codon site correlated with MAF. The most correlated with MAF was mutated codon site, but neither MAF nor mutated codon site correlated with recurrence. BMI, Body mass index; ECOG PS, The Eastern Cooperative Oncology Group performance score; MAF, Mutant allele frequency; pN, Pathological N factor; pT, Pathological T factor.

Correlation coefficients for each of the clinicopathologic factors, including MAF, were calculated using Python to create a heat map (Figure 2). The combinations with absolute correlation coefficients greater than 0.4 were mutated codon site and MAF, neoadjuvant therapy and cancer histology, neoadjuvant therapy and operating time, neoadjuvant therapy and intraoperative bleeding, adjuvant therapy and N‐factors, adjuvant therapy and stage, T factor and tumor diameter, T factor and stage, N factor and stage, operative time and intraoperative bleeding, preoperative MAF (Pre‐MAF) and MAF on postoperative day 1 (POD1‐MAF), Pre‐MAF and POD7‐MAF, Pre‐MAF and POD30‐MAF, POD1‐MAF and POD7‐MAF, POD1‐MAF and POD30‐MAF, and POD7‐MAF and POD30‐MAF. MAFs with different dates of blood sampling correlated with each other; otherwise, only mutated codon site correlated with MAF. No factor correlated with recurrence with a correlation coefficient of 0.4 or higher. As shown in Figure 2, the most correlated with recurrence was lymph node metastasis, followed by tumor T‐factor. On the other hand, the most correlated with MAF was mutated codon site, but neither MAF nor mutated codon site correlated with recurrence.

The clinicopathologic factors of the 25 patients with recurrence are summarized in Table 2. In this patient group, 60% had zero MAF at 30 days postoperatively.

**TABLE 2:** Clinicopathological factors in 25 CRC patients with recurrence.

Case	Sex	Age	Mutation	Primary location	Tumor size (mm)	Histological grade	Tumor depth	ly	v	Lymph node	Stage	Recurrence site	Time to recurrence (month)	MAF	MAF	MAF	MAF
														Preoperative	POD1	POD7	POD30
1	F	69	*BRAF*‐V600E	Ascending	40	Well	T4a	−	+	N2b	StageIIIc	Peritoneum	4	0.98	1.932	0	1.504
2	M	58	*BRAF*‐V600E	Ascending	40	Poorly	T4a	+	+	N2b	StageIIIc	Liver	3	0.103	0.512	0.248	0.621
3	M	52	*BRAF*‐V600E	Ascending	65	Moderately	T4a	+	+	N1b	StageIIIb	Peritoneum	5	4.878	0.854	4.371	1.875
4	F	77	*BRAF*‐V600E	Descending	35	Well	T4a	+	+	N0	StageIIb	Peritoneum	7	0.686	0.844	0.213	0.221
5	M	67	*KRAS*‐A146T	Rectum	80	Mucinous	T3	−	−	N0	StageIIa	Local	24	2.1	0.732	1.101	9.067
6	F	82	*KRAS*‐A146T	Rectum	55	Mucinous	T4a	+	+	N2a	StageIIIc	Lymph node	12	2.336	2.353	2.067	3.331
7	F	31	*KRAS*‐A146T	Rectum	30	Well	T3	+	−	N0	StageIIa	Lung	6	29.129	5.038	4.429	25.237
8	F	75	*KRAS*‐G12A	Rectum	105	Well	T3	+	+	N1b	StageIIIb	Lung	3	0	0	0	0
9	F	55	*KRAS*‐G12D	Descending	55	Well	T4a	+	+	N0	StageIIb	Liver	6	0	0	0	0.471
10	F	64	*KRAS*‐G12D	Rectum	40	Well	T3	+	+	N2a	StageIIIb	Liver, Lung	8	0.353	0	0	0
11	F	70	*KRAS*‐G12D	Sigmoid	60	Well	T4a	+	+	N0	StageIIa	Local, Peritoneum	8	0	0.63	0	0
12	F	76	*KRAS*‐G12D	Sigmoid	50	Well	T3	−	+	N0	StageIIa	Peritoneum	22	0.701	0.287	0	0.419
13	F	83	*KRAS*‐G12D	Rectum	25	Well	T2	+	−	N0	StageI	Lung	6	1.444	0	0	0
14	M	66	*KRAS*‐G12D	Rectum	60	Well	T3	−	+	N0	StageIIa	Lung	38	0	0	0	0
15	M	56	*KRAS*‐G12D	Ascending	35	Well	T3	+	+	N1b	StageIIIb	Lung	32	1.028	0	0	0
16	M	71	*KRAS*‐G12D	Descending	70	Mucinous	T3	+	+	N1b	StageIIIb	Lymph node	42	0	0	0.485	0.749
17	F	77	*KRAS*‐G12D	Ascending	35	Well	T3	+	+	N1b	StageIIIb	Liver	4	0	0.655	0	0
			*KRAS‐*G13D											0	0	0	0
18	M	69	*KRAS*‐G12S	Sigmoid	80	Well	T3	−	+	N0	StageIIa	Liver	7	3.433	0	0	0
19	M	80	*KRAS*‐G12S	Rectum	25	Well	T3	+	+	N0	StageIIa	Lung	15	0	0	0	0
20	M	60	*KRAS*‐G12V	Transverse	70	Well	T3	+	+	N1b	StageIIIb	Liver, Lung	14	0.959	0	0	0
21	M	49	*KRAS*‐G12V	Rectum	25	Well	T2	+	+	N0	StageI	Lung	21	0	0	0	0
22	M	60	*KRAS*‐G13D	Rectum	70	Well	T4a	+	+	N2a	StageIIIc	Peritoneum	11	0	0	0	0
23	M	68	*KRAS*‐G13D	Rectum	30	Moderately	T3	+	+	N2b	StageIIIc	Lung	18	2.348	0	0	0
24	M	62	*KRAS*‐Q61H	Ascending	40	Moderately	T4a	+	+	N2a	StageIIIc	Lymph node	13	0	3.804	3.841	2.464
25	M	65	*NRAS*‐Q61L	Ascending	50	Well	T4a	+	+	N1a	StageIIIa	Liver	5	0	0	0	0

*Note:* Of the 25 recurrent patients, 15 (60%) had 0% MAF at 30 days postoperatively. MAF was higher in patients with the *KRAS* A146T mutation compared to other mutations.

Abbreviations: F, female; M, male; MAF, mutant allele frequency; POD, postoperative day.

### 3.3 Evaluation of MAF Classified by Mutation Site

**FIGURE 3:**
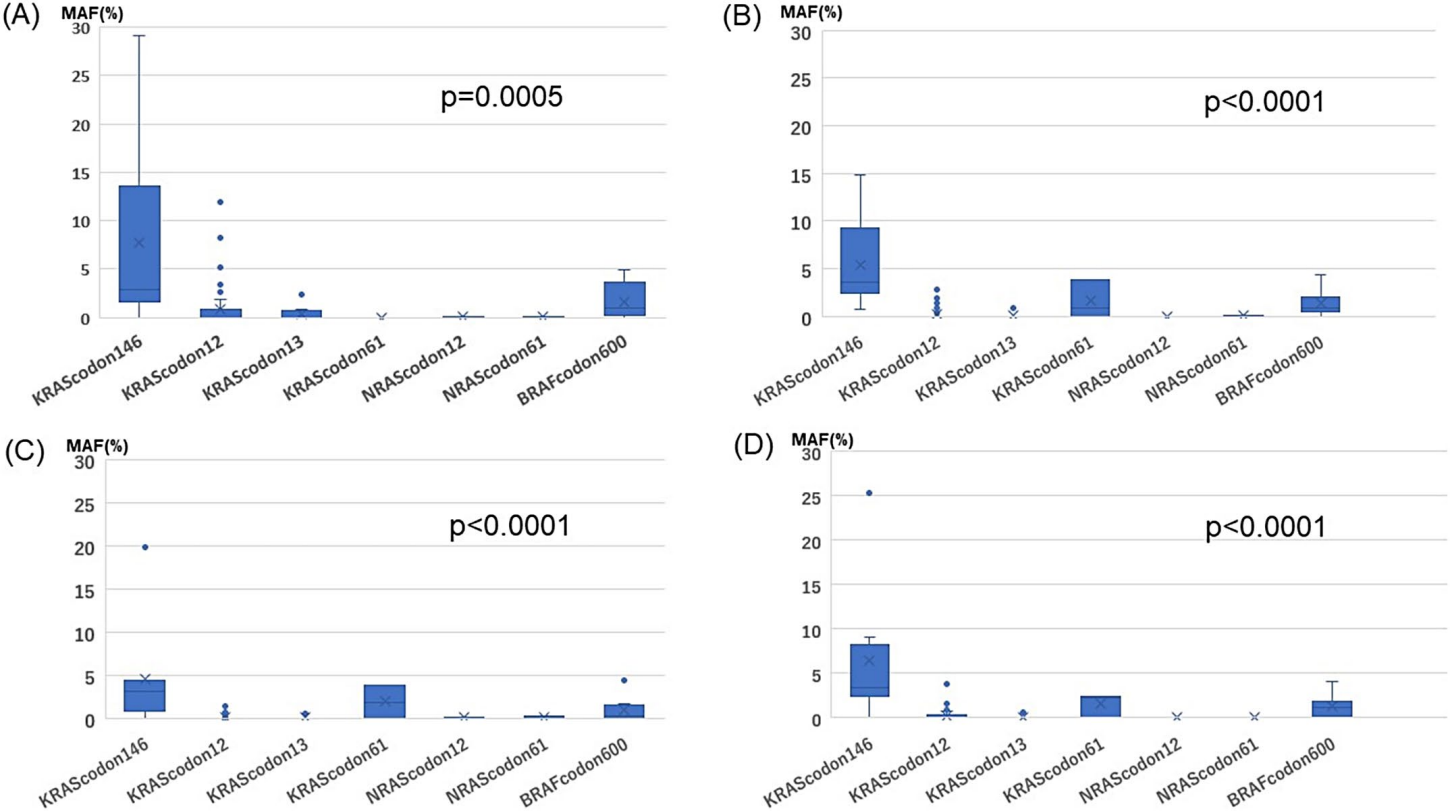
Comparison of MAF classified by mutation site. The relationship between perioperative MAF and mutation codons is shown separately for preoperative and postoperative days. The Kruskal‐Wallis test showed that there were significant differences between groups of mutation sites. (A) Preoperative MAF. (B) POD1 MAF. (C) POD7 MAF. (D) POD30 MAF. MAF, Mutant allele frequency; POD: Postoperative day.

Preoperative (Figure 3A), postoperative day 1 (Figure 3B), postoperative day 7 (Figure 3C), and postoperative day 30 (Figure 3D) MAFs were analyzed in accordance with mutated codon sites by statistical testing. Kruskal–Wallis analysis showed that there were significant differences between mutated codon sites at all MAF assessment time points. The MAF values of *KRAS* codon 146 at all time points were significantly higher than those of the other codons (*p* = 0.0005, *p* < 0.0001, *p* < 0.0001, *p* < 0.0001). In addition, Steel‐Dwass tests were performed for differences in MAF at the two mutated codon sites, and only results that were significantly different are shown in Table 3. *KRAS* codon 146 had significantly higher MAF values than *KRAS* codons 12 and 13 on all blood collection dates. Similarly, *BRAF* codon 600 had significantly higher MAF values than *KRAS* codon 12 on all blood collection dates.

**TABLE 3:** Comparison of MAF between the two groups classified by mutation site.

Date of blood sampling	Two mutated codons for comparison	*p*
Preoperative	*KRAS*codon146‐*KRAS*codon12	< 0.01
	*KRAS*codon146‐*KRAS*codon13	< 0.01
	*KRAS*codon146‐*KRAS*codon61	0.037
	*BRAF*codon600‐*KRAS*codon12	< 0.01
	*BRAF*codon600‐*KRAS*codon13	0.015
	*BRAF*codon600‐*KRAS*codon61	0.022
	*BRAF*codon600‐*NRAS*codon61	0.029
POD1	*KRAS*codon146‐*KRAS*codon12	< 0.01
	*KRAS*codon146‐*KRAS*codon13	< 0.01
	*BRAF*codon600‐*KRAS*codon12	< 0.01
POD7	*KRAS*codon146‐*KRAS*codon12	< 0.01
	*KRAS*codon146‐*KRAS*codon13	0.015
	*BRAF*codon600‐*KRAS*codon12	< 0.01
POD30	*KRAS*codon146‐*KRAS*codon12	< 0.01
	*KRAS*codon146‐*KRAS*codon13	< 0.01
	*BRAF*codon600‐*KRAS*codon12	< 0.01
	*BRAF*codon600‐*KRAS*codon13	< 0.01

*Note:* Steel‐Dwass tests were performed for differences in MAF at the two mutated codon sites, and only results that were significantly different are shown.

Abbreviations: MAF, mutant allele frequency; POD, postoperative day.

### 3.4 Evaluation of MAF Classified by Mutations

**FIGURE 4:**
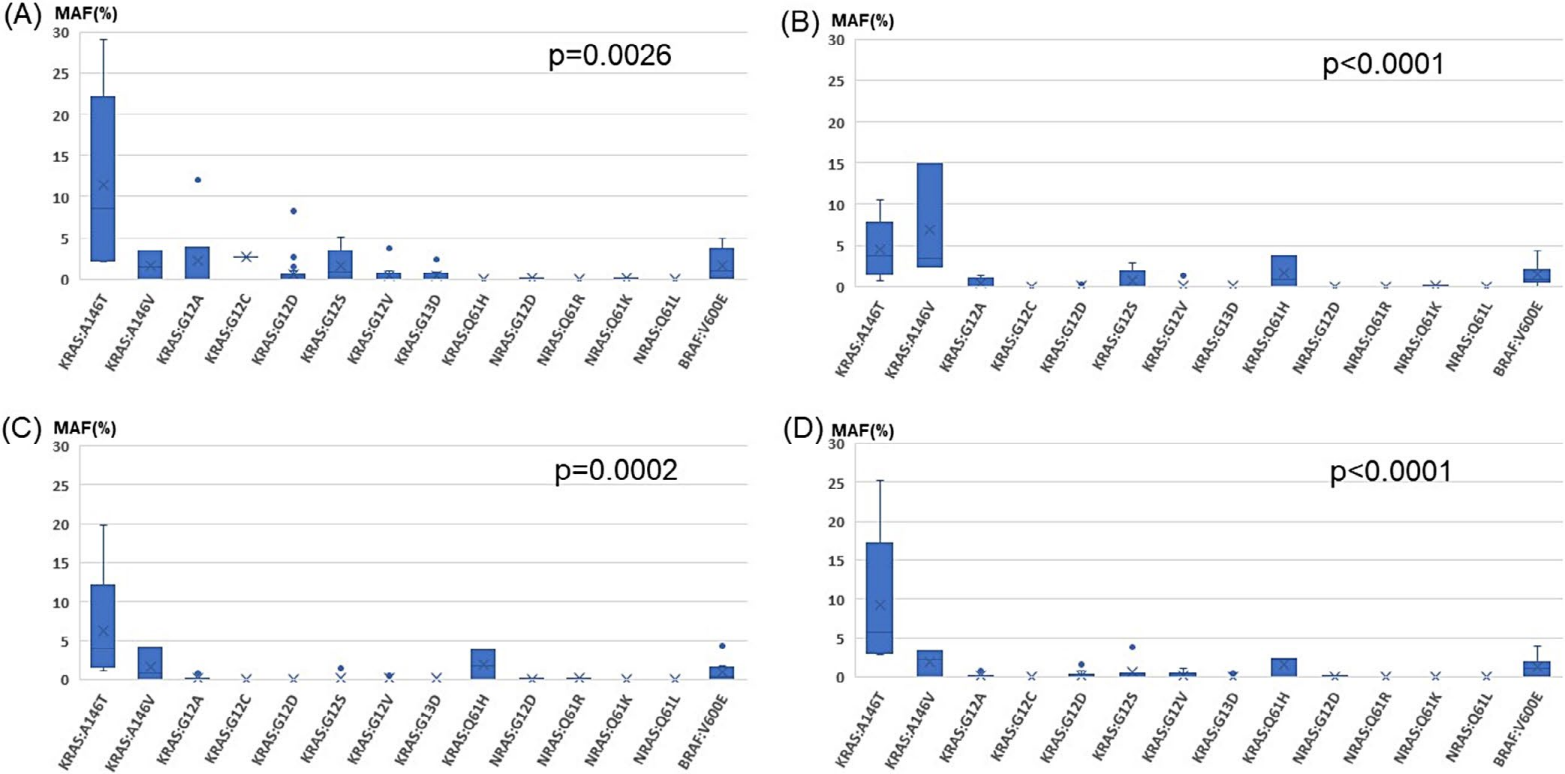
Comparison of MAF classified by mutation. The relationship between perioperative MAF and mutated amino acids is shown separately for preoperative and postoperative days. The Kruskal‐Wallis test showed that there were significant differences between groups of mutation sites. (A) Preoperative MAF. (B) POD1 MAF. (C) POD7 MAF. (D) POD30 MAF. MAF, Mutant allele frequency; POD, Postoperative day.

We next compared MAFs classified by mutations. Preoperative, postoperative day 1, postoperative day 7, and postoperative day 30 MAFs are shown in Figure 4A–D, respectively. Kruskal–Wallis analysis showed there was a significant difference in MAF values depending on the mutation at each MAF assessment time point (*p* = 0.0026, *p* < 0.0001, *p* = 0.0002, *p* < 0.0001). In addition, Steel‐Dwass tests were performed for differences in MAF at the two mutated amino acids, and only results that were significantly different are shown in Table 4. *KRAS* A146T had significantly higher MAF values than *KRAS* G12D on all blood collection dates. With regard to *NRAS* gene statistics, statistical analysis was not possible because there were only 1–2 cases each of G12D/Q61K/Q61L/Q61R mutations.

**TABLE 4:** Comparison of MAF between the two groups classified by mutated amino acids.

Date of blood sampling	Two mutated codons for comparison	*p*
Preoperative	*KRAS*codon146‐*KRAS*codon12	< 0.01
	*KRAS*codon146‐*KRAS*codon13	< 0.01
	*KRAS*codon146‐*KRAS*codon61	0.037
	*BRAF*codon600‐*KRAS*codon12	< 0.01
	*BRAF*codon600‐*KRAS*codon13	0.015
	*BRAF*codon600‐*KRAS*codon61	0.022
	*BRAF*codon600‐*NRAS*codon61	0.029
POD1	*KRAS*codon146‐*KRAS*codon12	< 0.01
	*KRAS*codon146‐*KRAS*codon13	< 0.01
	*BRAF*codon600‐*KRAS*codon12	< 0.01
POD7	*KRAS*codon146‐*KRAS*codon12	< 0.01
	*KRAS*codon146‐*KRAS*codon13	0.015
	*BRAF*codon600‐*KRAS*codon12	< 0.01
POD30	*KRAS*codon146‐*KRAS*codon12	< 0.01
	*KRAS*codon146‐*KRAS*codon13	< 0.01
	*BRAF*codon600‐*KRAS*codon12	< 0.01
	*BRAF*codon600‐*KRAS*codon13	< 0.01

*Note:* Steel‐Dwass tests were performed for differences in MAF at the two mutated amino acids, and only results that were significantly different are shown.

Abbreviations: MAF, mutant allele frequency; POD, postoperative day.

## 4 Discussion

Recent advancements in ctDNA assays have provided a means to detect minimal residual disease following curative surgery, offering the potential to revolutionize recurrence risk assessment and adjuvant chemotherapy protocols [25, 26, 27, 28]. However, identifying the genetic mutations to target remains unclear. Studies have shown that the dissemination of cancer cells into the bloodstream from the primary tumor occurs in the early stages of cancer, but the process of establishing metastasis is quite inefficient [29, 30, 31, 32, 33]. As the presence of cancer cells in the blood does not equate to recurrence, the identification of specific genetic mutations associated with recurrence is crucial for the accurate assessment of recurrence. Our previous study found that *KRAS* and *BRAF* mutations in ctDNA are not predictive of cancer relapse [19, 20], suggesting that the use of these mutations in ctDNA alone is insufficient for recurrence prediction. A number of studies have investigated the correlation between *KRAS* mutations in ctDNA and recurrence following radical resection of colorectal cancer [23, 34, 35, 36, 37]. However, several factors, including the stage of CRC, the *KRAS* mutant gene targets, the ctDNA detection method, the cutoff value, and the timing of blood collection, vary among these studies. Therefore, it is necessary to consider a variety of factors when examining the relationship between MAF and recurrence. As shown in Figure 2, the mutated codons were the only ones that correlated with MAFs for multiple dates. The strength of this study is that it showed that the percentage of MAFs detected differed depending on the mutation codon. No reports have examined the relationship between MAF and *KRAS* gene mutation sites.

Several reports have examined the utility of ctDNA for the early detection of recurrence after radical resection of colorectal cancer [19, 38, 39, 40, 41, 42, 43, 44, 45, 46, 47, 48, 49, 50, 51, 52]. The studies examined MAF cutoff values for each gene, but no studies have set cutoff values for each mutation site. Faulkner et al. reported a meta‐analysis of the utility of ctDNA in detecting minimal residual disease following curative surgery in colorectal cancer [53]. The ctDNA detection rate after colorectal cancer surgery varied widely from 4.2% to 90.9%, and the ctDNA detection rate in recurrent cases also varied widely from 23.8% to 100%. This is because of differences in ctDNA detection methods and the setting of cutoff values. Cutoff values vary because each study sets a value that is significantly different in the specific target population, and some studies do not publish cutoff values. In this study, we examined the correlation coefficients, shown in Table 2, and found a correlation between the gene mutation site and MAF. Further examination revealed that even in the same gene, MAF values differed greatly depending on the site of mutation. We believe that MAF cutoff values need to be established not only for each gene but also for each mutation site.

This study has several limitations. First, the sample size was not large, and data were obtained from a single institution. Therefore, a multicenter study is needed to increase the number of patients. Second, the observation period of this study was only 30 months, and thus other patients may relapse in the future. We believe that further long‐term follow‐up surveys should be conducted and reported in the future. Third, only *KRAS*, *NRAS*, and *BRAF* mutations were examined in this study. Cancer is spatially and temporally heterogeneous, and it is possible that recurrent tumors may not carry these gene mutations. Future studies should examine more genetic mutations directly associated with recurrence to predict recurrence.

## Author Contributions

**Fumihiro Yoshimura:** data analysis, writing of the manuscript, and editing and review of manuscript drafts. **Yoichiro Yoshida:** conceptualization and design, administrative support, provision of study materials and/or patients, collection and assembly of data, and data analysis and interpretation. **Teppei Yamada:** data collection and editing and review of manuscript drafts. **Keita Tanaka:** provision of research materials and/or patients and collection and assembly of data. **Takaomi Hayashi:** provision of analysis equipment, data collection. **Hideki Shimaoka:** provision of research materials, collection and tabulation of data. **Ryohei Sakamoto:** provision of study materials and/or patients and collection and assembly of data. **Naoya Aisu:** provision of research materials, collection and tabulation of data. **Gumpei Yoshimatsu:** data analysis and interpretation. **Suguru Hasegawa:** data analysis and interpretation.

## Ethics Statement

The Institutional Review Board of Fukuoka University Faculty of Medicine approved this research (U19.09.001, 2017‐M‐35).

## Conflicts of Interest

The authors declare no conflicts of interest.

## Data Availability

The datasets analyzed in this study are available from the corresponding author upon request.

## References

[1] H. Sung , J. Ferlay , R. L. Siegel , et al., “Global Cancer Statistics 2020: GLOBOCAN Estimates of Incidence and Mortality Worldwide for 36 Cancers in 185 Countries,” CA: A Cancer Journal for Clinicians 71 (2021): 209–249. 33538338 10.3322/caac.21660

[2] E. Osterman and B. Glimelius , “Recurrence Risk After Up‐To‐Date Colon Cancer Staging, Surgery, and Pathology: Analysis of the Entire Swedish Population,” Diseases of the Colon & Rectum 61 (2018): 1016–1025. 30086050 10.1097/DCR.0000000000001158

[3] C. Tan and X. Du , “KRAS Mutation Testing in Metastatic Colorectal Cancer,” World Journal of Gastroenterology: WJG 18 (2012): 5171. 23066310 10.3748/wjg.v18.i37.5171 PMC3468848

[4] C. P. Vaughn , S. D. ZoBell , L. V. Furtado , C. L. Baker , and W. S. Samowitz , “Frequency of KRAS, BRAF, and NRAS Mutations in Colorectal Cancer,” Genes, Chromosomes & Cancer 50 (2011): 307–312. 21305640 10.1002/gcc.20854

[5] P. A. Myer , J. K. Lee , R. W. Madison , et al., “The Genomics of Colorectal Cancer in Populations With African and European AncestryGenomics, Colorectal Cancer, and African and European Ancestry,” Cancer Discovery 12 (2022): 1282–1293. 35176763 10.1158/2159-8290.CD-21-0813 PMC9169495

[6] D. Vigil , J. Cherfils , K. L. Rossman , and C. J. Der , “Ras Superfamily GEFs and GAPs: Validated and Tractable Targets for Cancer Therapy?,” Nature Reviews Cancer 10 (2010): 842–857. 21102635 10.1038/nrc2960 PMC3124093

[7] H. Lavoie and M. Therrien , “Regulation of RAF Protein Kinases in ERK Signalling,” Nature Reviews. Molecular Cell Biology 16 (2015): 281–298, 10.1038/nrm3979. 25907612

[8] M. Gerlinger , A. J. Rowan , S. Horswell , et al., “Intratumor Heterogeneity and Branched Evolution Revealed by Multiregion Sequencing,” New England Journal of Medicine 366 (2012): 883–892. 22397650 10.1056/NEJMoa1113205 PMC4878653

[9] X. Han , J. Wang , and Y. Sun , “Circulating Tumor DNA as Biomarkers for Cancer Detection,” Genomics, Proteomics & Bioinformatics 15 (2017): 59–72. 10.1016/j.gpb.2016.12.004 PMC5414889 28392479

[10] M. Urbini , G. Marisi , I. Azzali , et al., “Dynamic Monitoring of Circulating Tumor DNA in Patients With Metastatic Colorectal Cancer,” JCO Precision Oncology 7 (2023): e2200694, 10.1200/po.22.00694. 37656949

[11] J. T. Topham , C. J. O'Callaghan , H. Feilotter , et al., “Circulating Tumor DNA Identifies Diverse Landscape of Acquired Resistance to Anti‐Epidermal Growth Factor Receptor Therapy in Metastatic Colorectal Cancer,” Journal of Clinical Oncology 41 (2023): 485–496, 10.1200/jco.22.00364. 36007218 PMC9870216

[12] C. Bettegowda , M. Sausen , R. J. Leary , et al., “Detection of Circulating Tumor DNA in Early‐and Late‐Stage Human Malignancies,” Science Translational Medicine 6 (2014): 224ra224. 10.1126/scitranslmed.3007094 PMC4017867 24553385

[13] L. A. Diaz, Jr. and A. Bardelli , “Liquid Biopsies: Genotyping Circulating Tumor DNA,” Journal of Clinical Oncology 32 (2014): 579–586. 24449238 10.1200/JCO.2012.45.2011 PMC4820760

[14] T. Yamada , T. Iwai , G. Takahashi , et al., “Utility of KRAS Mutation Detection Using Circulating Cell‐Free DNA From Patients With Colorectal Cancer,” Cancer Science 107 (2016): 936–943. 27116474 10.1111/cas.12959 PMC4946708

[15] F. Sclafani , I. Chau , D. Cunningham , et al., “KRAS and BRAF Mutations in Circulating Tumour DNA From Locally Advanced Rectal Cancer,” Scientific Reports 8 (2018): 1–9. 29362371 10.1038/s41598-018-19212-5 PMC5780472

[16] R. Ohta , T. Yamada , H. Sonoda , et al., “Detection of KRAS Mutations in Circulating Tumour DNA From Plasma and Urine of Patients With Colorectal Cancer,” European Journal of Surgical Oncology 47 (2021): 3151–3156. 34315643 10.1016/j.ejso.2021.07.017

[17] M. C. Maia , M. Salgia , and S. K. Pal , “Harnessing Cell‐Free DNA: Plasma Circulating Tumour DNA for Liquid Biopsy in Genitourinary Cancers,” Nature Reviews Urology 17 (2020): 271–291. 32203306 10.1038/s41585-020-0297-9

[18] M. Russano , A. Napolitano , G. Ribelli , et al., “Liquid Biopsy and Tumor Heterogeneity in Metastatic Solid Tumors: The Potentiality of Blood Samples,” Journal of Experimental & Clinical Cancer Research 39 (2020): 1–13. 32460897 10.1186/s13046-020-01601-2 PMC7254767

[19] K. Tanaka , Y. Yoshida , T. Yamada , et al., “Oncological Evaluation in the Perioperative Period Using cfDNA With BRAF V600E Mutation in Patients With Colorectal Cancer,” Scientific Reports 11 (2021): 1–8. 34168268 10.1038/s41598-021-92795-8 PMC8225636

[20] T. Hayashi , Y. Yoshida , T. Yamada , et al., “Relationship Between Perioperative Oncological Evaluation and Recurrence Using Circulating Tumor DNA With KRAS Mutation in Patients With Colorectal Cancer,” Cancer Medicine 11 (2022): 3126–3135. 35312176 10.1002/cam4.4677 PMC9385586

[21] N. Verschoor , M. K. Bos , E. de Oomen‐ Hoop , et al., “A Review of Trials Investigating ctDNA‐Guided Adjuvant Treatment of Solid Tumors: The Importance of Trial Design,” European Journal of Cancer (Oxford, England: 1990) 207 (2024): 114159, 10.1016/j.ejca.2024.114159. 38878446

[22] K. H. Lee , T. H. Lee , M. K. Choi , I. S. Kwon , G. E. Bae , and M. K. Yeo , “Identification of a Clinical Cutoff Value for Multiplex KRASG12/G13 Mutation Detection in Colorectal Adenocarcinoma Patients Using Digital Droplet PCR, and Comparison With Sanger Sequencing and PNA Clamping Assay,” Journal of Clinical Medicine 9 (2020): 2283. 32708359 10.3390/jcm9072283 PMC7409004

[23] Y. Nakamura , S. Yokoyama , K. Matsuda , et al., “Preoperative Detection of KRAS Mutated Circulating Tumor DNA Is an Independent Risk Factor for Recurrence in Colorectal Cancer,” Scientific Reports 11 (2021): 1–8. 33432066 10.1038/s41598-020-79909-4 PMC7801374

[24] A. Perdyan , P. Spychalski , J. Kacperczyk , O. Rostkowska , and J. Kobiela , “Circulating Tumor DNA in KRAS Positive Colorectal Cancer Patients as a Prognostic Factor–A Systematic Review and Meta‐Analysis,” Critical Reviews in Oncology/Hematology 154 (2020): 103065. 32763752 10.1016/j.critrevonc.2020.103065

[25] J. Tie , Y. Wang , C. Tomasetti , et al., “Circulating Tumor DNA Analysis Detects Minimal Residual Disease and Predicts Recurrence in Patients With Stage II Colon Cancer,” Science Translational Medicine 8 (2016): 346ra392. 10.1126/scitranslmed.aaf6219 PMC5346159 27384348

[26] R. B. Corcoran and B. A. Chabner , “Application of Cell‐Free DNA Analysis to Cancer Treatment,” New England Journal of Medicine 379 (2018): 1754–1765. 30380390 10.1056/NEJMra1706174

[27] K. J. Thomas Craig , V. C. Willis , D. Gruen , K. Rhee , and G. P. Jackson , “The Burden of the Digital Environment: A Systematic Review on Organization‐Directed Workplace Interventions to Mitigate Physician Burnout,” Journal of the American Medical Informatics Association 28 (2021): 985–997. 33463680 10.1093/jamia/ocaa301 PMC8068437

[28] Y. Wang , L. Li , J. D. Cohen , et al., “Prognostic Potential of Circulating Tumor DNA Measurement in Postoperative Surveillance of Nonmetastatic Colorectal Cancer,” JAMA Oncology 5 (2019): 1118–1123. 31070668 10.1001/jamaoncol.2019.0512 PMC6512291

[29] H. Patel , N. le Marer , R. Q. Wharton , et al., “Clearance of Circulating Tumor Cells After Excision of Primary Colorectal Cancer,” Annals of Surgery 235 (2002): 226–231. 11807362 10.1097/00000658-200202000-00010 PMC1422418

[30] J. Phallen , M. Sausen , V. Adleff , et al., “Direct Detection of Early‐Stage Cancers Using Circulating Tumor DNA,” Science Translational Medicine 9 (2017): eaan2415. 28814544 10.1126/scitranslmed.aan2415 PMC6714979

[31] L. Weiss , “Metastatic Inefficiency,” Advances in Cancer Research 54 (1990): 159–211. 1688681 10.1016/s0065-230x(08)60811-8

[32] I. J. Fidler , “Metastasis: Quantitative Analysis of Distribution and Fate of Tumor Emboli Labeled With 125I‐5‐Iodo‐2′‐Deoxyuridine,” Journal of the National Cancer Institute 45 (1970): 773–782. 5513503

[33] K. J. Luzzi , I. C. MacDonald , E. E. Schmidt , et al., “Multistep Nature of Metastatic Inefficiency: Dormancy of Solitary Cells After Successful Extravasation and Limited Survival of Early Micrometastases,” American Journal of Pathology 153 (1998): 865–873. 9736035 10.1016/S0002-9440(10)65628-3 PMC1853000

[34] T. Lecomte , A. Berger , F. Zinzindohoué , et al., “Detection of Free‐Circulating Tumor‐Associated DNA in Plasma of Colorectal Cancer Patients and Its Association With Prognosis,” International Journal of Cancer 100 (2002): 542–548. 12124803 10.1002/ijc.10526

[35] B. Ryan , F. Lefort , R. McManus , et al., “A Prospective Study of Circulating Mutant KRAS2 in the Serum of Patients With Colorectal Neoplasia: Strong Prognostic Indicator in Postoperative Follow Up,” Gut 52 (2003): 101–108. 12477769 10.1136/gut.52.1.101 PMC1773535

[36] L. V. Schøler , T. Reinert , M. B. W. Ørntoft , et al., “Clinical Implications of Monitoring Circulating Tumor DNA in Patients With Colorectal Cancer,” Clinical Cancer Research 23 (2017): 5437–5445. 28600478 10.1158/1078-0432.CCR-17-0510

[37] C. E. B. Thomsen , A. L. Appelt , R. F. Andersen , J. Lindebjerg , L. H. Jensen , and A. Jakobsen , “The Prognostic Value of Simultaneous Tumor and Serum RAS/RAF Mutations in Localized Colon Cancer,” Cancer Medicine 6 (2017): 928–936. 28378527 10.1002/cam4.1051 PMC5430097

[38] A. Grancher , L. Beaussire , S. Manfredi , et al., “Postoperative Circulating Tumor DNA Detection Is Associated With the Risk of Recurrence in Patients Resected for a Stage II Colorectal Cancer,” Frontiers in Oncology 12 (2022): 973167, 10.3389/fonc.2022.973167. 36439476 PMC9685416

[39] M. Allegretti , G. Cottone , F. Carboni , et al., “Cross‐Sectional Analysis of Circulating Tumor DNA in Primary Colorectal Cancer at Surgery and During Post‐Surgery Follow‐Up by Liquid Biopsy,” Journal of Experimental & Clinical Cancer Research 39 (2020): 1–12. 32312295 10.1186/s13046-020-01569-z PMC7168847

[40] P. Carpinetti , E. Donnard , F. Bettoni , et al., “The Use of Personalized Biomarkers and Liquid Biopsies to Monitor Treatment Response and Disease Recurrence in Locally Advanced Rectal Cancer After Neoadjuvant Chemoradiation,” Oncotarget 6 (2015): 38360–38371, 10.18632/oncotarget.5256. 26451609 PMC4742005

[41] G. Chen , J. Peng , Q. Xiao , et al., “Postoperative Circulating Tumor DNA as Markers of Recurrence Risk in Stages II to III Colorectal Cancer,” Journal of Hematology & Oncology 14 (2021): 80, 10.1186/s13045-021-01089-z. 34001194 PMC8130394

[42] D. Ji , D. Zhang , T. Zhan , et al., “Tumor Mutation Burden in Blood Predicts Benefit From Neoadjuvant Chemo/Radiotherapy in Locally Advanced Rectal Cancer,” Genomics 113 (2021): 957–966, 10.1016/j.ygeno.2020.10.029. 33129922

[43] S. Khakoo , P. D. Carter , G. Brown , et al., “MRI Tumor Regression Grade and Circulating Tumor DNA as Complementary Tools to Assess Response and Guide Therapy Adaptation in Rectal Cancer,” Clinical Cancer Research 26 (2020): 183–192, 10.1158/1078-0432.Ccr-19-1996. 31852830

[44] U. Lindforss , H. Zetterquist , N. Papadogiannakis , and H. Olivecrona , “Persistence of K‐Ras Mutations in Plasma After Colorectal Tumor Resection,” Anticancer Research 25 (2005): 657–661. 15816642

[45] S. Murahashi , T. Akiyoshi , T. Sano , et al., “Serial Circulating Tumour DNA Analysis for Locally Advanced Rectal Cancer Treated With Preoperative Therapy: Prediction of Pathological Response and Postoperative Recurrence,” British Journal of Cancer 123 (2020): 803–810, 10.1038/s41416-020-0941-4. 32565539 PMC7462982

[46] S. B. Ng , C. Chua , M. Ng , et al., “Individualised Multiplexed Circulating Tumour DNA Assays for Monitoring of Tumour Presence in Patients After Colorectal Cancer Surgery,” Scientific Reports 7 (2017): 40737, 10.1038/srep40737. 28102343 PMC5244357

[47] T. Reinert , T. V. Henriksen , E. Christensen , et al., “Analysis of Plasma Cell‐Free DNA by Ultradeep Sequencing in Patients With Stages I to III Colorectal Cancer,” JAMA Oncology 5 (2019): 1124–1131, 10.1001/jamaoncol.2019.0528. 31070691 PMC6512280

[48] T. Suzuki , T. Suzuki , Y. Yoshimura , et al., “Detection of Circulating Tumor DNA in Patients of Operative Colorectal and Gastric Cancers,” Oncotarget 11 (2020): 3198–3207, 10.18632/oncotarget.27682. 32922660 PMC7456613

[49] J. Taieb , V. Taly , J. Henriques , et al., “Prognostic Value and Relation With Adjuvant Treatment Duration of ctDNA in Stage III Colon Cancer: A Post Hoc Analysis of the PRODIGE‐GERCOR IDEA‐France Trial,” Clinical Cancer Research 27 (2021): 5638–5646, 10.1158/1078-0432.Ccr-21-0271. 34083233

[50] N. Tarazona , F. Gimeno‐Valiente , V. Gambardella , et al., “Targeted Next‐Generation Sequencing of Circulating‐Tumor DNA for Tracking Minimal Residual Disease in Localized Colon Cancer,” Annals of Oncology 30 (2019): 1804–1812, 10.1093/annonc/mdz390. 31562764

[51] J. Tie , J. D. Cohen , Y. Wang , et al., “Circulating Tumor DNA Analyses as Markers of Recurrence Risk and Benefit of Adjuvant Therapy for Stage III Colon Cancer,” JAMA Oncology 5 (2019): 1710–1717, 10.1001/jamaoncol.2019.3616. 31621801 PMC6802034

[52] J. Zhou , C. Wang , G. Lin , et al., “Serial Circulating Tumor DNA in Predicting and Monitoring the Effect of Neoadjuvant Chemoradiotherapy in Patients With Rectal Cancer: A Prospective Multicenter Study,” Clinical Cancer Research 27 (2021): 301–310, 10.1158/1078-0432.Ccr-20-2299. 33046514

[53] L. G. Faulkner , L. M. Howells , C. Pepper , J. A. Shaw , and A. L. Thomas , “The Utility of ctDNA in Detecting Minimal Residual Disease Following Curative Surgery in Colorectal Cancer: A Systematic Review and Meta‐Analysis,” British Journal of Cancer 128 (2023): 297–309, 10.1038/s41416-022-02017-9. 36347967 PMC9902552

